# Bees on the run: *Nosema* spp. (Microsporidia) in *Apis mellifera* and related products, Italy

**DOI:** 10.3389/fvets.2024.1530169

**Published:** 2025-01-06

**Authors:** Giovanni Sgroi, Luigi Jacopo D’Auria, Maria Gabriella Lucibelli, Andrea Mancusi, Yolande Thérèsa Rose Proroga, Mauro Esposito, Simona Rea, Daniel Signorelli, Federica Gargano, Nicola D’Alessio, Ranju Ravindran Santhakumari Manoj, Peyman Khademi, Giuseppe Rofrano

**Affiliations:** ^1^Experimental Zooprophylactic Institute of Southern Italy, Portici, Naples, Italy; ^2^Centro di Referenza Nazionale per l’analisi e lo studio delle correlazioni tra ambiente, animali e uomini, Portici, Naples, Italy; ^3^Department of Cultural Heritage Sciences, University of Salerno, Salerno, Italy; ^4^Department of Population Medicine and Diagnostics, Cornell University, Ithaca, NY, United States; ^5^Department of Microbiology and Food Hygiene, University of Lorestan, Khorramabad, Iran

**Keywords:** bee decline, honeybee, honey, pollen, Nosema, nosemosis, *Vairimorpha*, Italy

## Abstract

**Introduction:**

The decline of the European/western honeybee (*Apis mellifera*) population is on account of a plethora of microorganisms, such as *Nosema apis* and *Nosema ceranae*, two microsporidian fungi responsible of nosemosis that affects welfare and production of the bee industry. Accordingly, this study aimed to investigate the presence of both pathogens in bees, pollen and honey from apiaries in Southwestern Italy.

**Methods:**

From March to July 2022 and 2023, apiaries (*n* = 10) were selected and classified as High Impact Areas (HIAs, *n* = 5) and Low Impact Areas (LIAs, *n* = 5) according to a 5-point environmental risk index based on factors affecting bee health sand related productions. Bee, pollen and honey samples, were collected and tested for *Nosema* spp. DNA by specific PCR protocols targeting the 16S rRNA gene. Signs/symptoms of nosemosis were monitored and collected by the cooperation of beekeepers.

**Results:**

Out of 10 apiaries, 6 (i.e., 60%, 95% CI: 31.3–83.2) tested positive for at least one sample to *Nosema* spp. DNA, being 2 positives for *N. apis*, 2 for *N. ceranae* and 2 co-infected (i.e., 20%, 5.7–51.0). Based on the biological samples, honey was positive for *N. apis* in one apiary, pollen for *N. ceranae* in two apiaries, and bees for *N. apis* in 3 apiaries, *N. ceranae* in 1 apiary, and both species in 1 apiary. In all the apiaries positive to *N. apis* and *N. ceranae*, high mortality and low honey production were observed. A higher risk of infection was observed in apiaries from HIAs (*OR* = 6.00). The sequences of *N. apis* and *N. ceranae* had 99.5–100% homology with those in the GenBank database. Whereas all sequences of *N. apis* were identical to each other, four sequences types of *N. ceranae* characterized by single nucleotide polymorphisms (SNPs) were identified. The computation of polymorphisms revealed high haplotype diversity (i.e., *Hd* = 1.000) and low nucleotide diversity (i.e., *Pi* = 0.00913) of *N. ceranae* sequence types.

**Discussion:**

This study reveals a high circulation of *N. apis* and *N. ceranae* in Southwestern Italy, indicating the need for improved monitoring of these microsporidia to protect bee welfare and bee industry.

## 1 Introduction

In last years, a plethora of infectious and parasitic agents has affected the population decline of pollinating insects worldwide (1). Among these, the European honey bee (or western honey bee) *Apis mellifera* is commonly exposed to fungi of the genus *Nosema* spp. (phylum Microsporidia, family Nosematidae), obligate intracellular microsporidia that infect ventricular epithelium cells of adut bees causing nosemosis (2–5), mainly in high density apiaries (6). Nosemosis is one of the main causes of bee colony collapse and prodution loss for beekeepers (7) due to the severe dysentery caused in bees (8, 9). In addition, the oro-fecal transmission pathway of *Nosema* spp. and the typical food-sharing behaviour of bees (i.e., trophallaxis) (10) favour the circulation of microsporidian spores among bees, but also pollen and honey, causing organoleptic and production alterations in honey (11–15). To date, two different pathogenic species of *Nosema* spp. have been reported, *Nosema apis* (synonym *Vairimorpha apis*) and *Nosema ceranae* (synonym *Vairimorpha ceranae*) (16, 17). While *N. apis* is known from the beginning of the XX century (18), *N. ceranae* is an emerging species reported for the first time in 2005 (19) and now spread throughout Europe (20–26), as well as in Canada, USA, and south America (27–30).

In perspective, the fact that long cold winters, intense rain, and high relative humidity can favour the spread of *N. apis* and *N. ceranae* is of concern considering the running climatic changes and global warming (31–34), especially for *N. ceranae* that is considered replacing *N. apis* in several areas (35). As for Italy, *N. ceranae* has been reported in apiaries from central and Northern Italy, whereas the last report of *N. apis* dates back to 2010 in the North of the peninsula according to a nationwide 2 year-monitoring plan (36). Moreover, the occurrence of *N. apis* has been recently reported in apiaries of Southeastern Italy (15), suggesting the potential spread of this microsporidian also in other Southern areas of the country.

In order to verify this hypothesis, this study aimed to investigate the presence and distribution of *Nosema* spp. DNA in *A. mellifera* specimens and their products (i.e., pollen and honey) in selected apiaries of southwestern Italy.

## 2 Materials and methods

### 2.1 Study area and sampling

This study was approved by the Italian Ministry of Health within the project authorization no. IZS ME 08/22 RC aimed to assess the bee welfare and related productions of Southern Italy.

**Figure 1:**
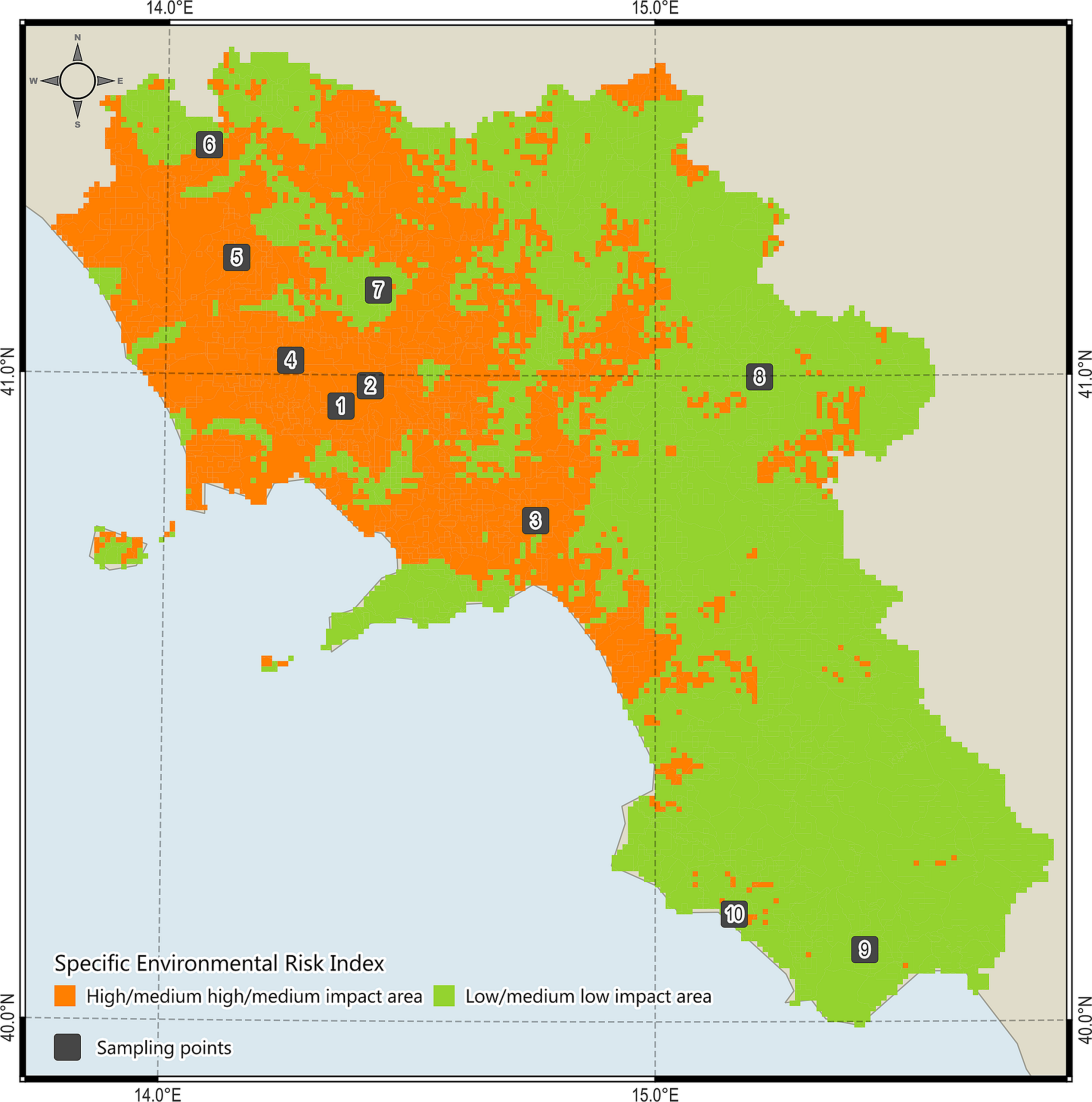
High impact areas (HIAs, *n* = 5) and low impact areas (LIAs, *n* = 5) investigated for *Nosema* and *Nosema* DNAs in the study area.

The study was carried out in the Campania region, Southern Italy, characterized by a typical Mediterranean temperate climate and progressively continental features of mainland and mountainous landscapes (37). In order to investigate aspects potentially correlated to presence of *Nosema* spp. and health status of bees, sampling areas were selected by using a risk index based on 5 environmental variables (i.e., pollution, land use, hydrographic network, air quality, bee density) and classified as high impact areas (HIAs) or low impact areas (LIAs) (38). Then, from March to July 2022–2023, bees, pollen and honey samples were collected in 10 different apiaries (5 LIAs and 5 HIAs) (Figure 1) by the staff of the Experimental Zooprophylactic Institute of Southern Italy (Portici, Italy) in collaboration with apiary owners that monitored any signs/symptoms of the hive potentially related to the infections. For each apiary, bee specimens, pollen and honey were collected using *under-basket* cages (39), combs inside the hives, and traps installed in front of the hives. For each apiary, a total of 50 bees, 10 g pollen and 1 g honey were collected from different hives, stored at −20°C into specific 500 mL glass jars labelled, and delivered to the Animal Health Department of the Experimental Zooprophylactic Institute of Southern Italy (Portici, Italy).

### 2.2 DNA extraction, PCR protocol, and sequencing

DNA was extracted from bee, pollen and honey samples using the QIAamp DNA mini-KIT tissue protocol (Qiagen, Hilden, Germany) according to the manufacturer’s instructions. The obtained DNA was diluted 1:10 with Tris-glycine TGBE buffer and centrifuged at 5,000 g for 5 min. For each sample, DNA concentration was determined by BioPhotometer plus biophotometer (Eppendorf AG, Hamburg, Germany). All DNAs were screened for *N. apis* and *N. ceranae* by using two species-specific endpoint PCR protocols targeting the 16S rRNA gene, according to the National Reference Laboratory for honeybee health (40); primer details are listed in Table 1. PCRs were performed in a total volume of 25 μL containing 12.5 μL HotStarTaq Master Mix, 0.625 μL of each primer at concentration of 0.25 μM, 9.25 μL of Dnase/Rnase free water and 2 μL of DNA template. Amplification conditions included an initial denaturation/activation step at 95°C for 15 min, followed by 35 cycles of denaturation at 94°C for 15 s, primers annealing at 62°C for 30s and extension at 72°C per 30s, and a final extension step at 72°C for 7 min. Amplicons were displayed by automated capillary electrophoresis with the QIAxcel instrument (Qiagen, Hilden, Germany). Amplicons were then purified with the QIAquick PCR Purification kit (Qiagen, Hilden, Germany) and sequenced in both directions using the same primers as for PCR, with the Big Dye Terminator Cycle Sequencing Kit v1.1 in the automated sequencer 3,500 Genetic Analyzer (Thermo Fischer Scientific, United States). Consensus sequences were aligned and edited *via* ClustalW Multiple Alignment method in the BioEdit software (version 7.7) (41), and compared with those available in the GenBank database with the Basic Local Alignment Search Tool (BLAST).^1^ Edited sequences were then exported to DnaSP software (version 6) (42) for the computation of polymorphisms using Tajima’s (43) and Fu’s Fs tests (44).

**Table 1:** Primers for the detection of *Nosema apis* and *Nosema ceranae* used in this study.

Pathogen	Primer	Sequence 5′–3’	Amplicon (bp)
*Nosema apis*	321APIS-FOR	GGGGGCATGTCTTTGACGTACTATGTA	321
	321APIS-REV	GGGGGGCGTTTAAAATGTGAAACAACTATG	
*Nosema ceranae*	218MITOC-FOR	CGGCGACGATGTGATATGAAAATATTAA	219
	218MITOC-REV	CCCGGTCATTCTCAAACAAAAAACCG	

All the sequences were submitted on GenBank database under the accession numbers PP758584 for *N. apis* and PP758589-PP758592 for *N. ceranae*, respectively.

### 2.3 Statistical analysis

Exact binomial 95% confidence intervals (95% CIs) by Wilson’s method were calculated for the proportions of infection herein found. The Fisher’s exact test was used for assessing any statistical differences in the frequency of infection according to the geographical origin of samples; a value of *p* < 0.05 was considered statistically significant. The Odds Ratio (*OR*) was used to verify the difference of infection risk according to the geographical origin of samples. Statistical analyses were performed by using the online software Epitools-Epidemiological Calculators (45). The distribution of *Nosema* spp.—positive samples in the study area was obtained with QGIS software (version 3.34.10-Prizren LTR).

## 3 Results

Out of 10 apiaries investigated, 6 (i.e., 60%, 95% CI: 31.3–83.2) tested positive for at least one sample to *Nosema* spp. DNA, being 2 positives only for *N. apis*, 2 only for *N. ceranae* and 2 co-infected (i.e., 20%, 5.7–51.0). Details of positivity to *N. apis* and *N. ceranae*, according to the different samples examined (i.e., bee specimen, pollen and honey) and apiary typology (i.e., HIAs or LIAs), are reported in Table 2. In all the apiaries tested positive for *N. apis* and *N. ceranae*, high mortality and low honey production were observed.

**Table 2:** Bee farms tested negative (Neg) and positive to *Nosema* spp. DNA, according to different samples (i.e., bee specimens, honey, pollen).

Apiary	Typology	Bees	Pollen	Honey
1	HIAs	Neg	*N. ceranae*	Neg
2	HIAs	*N. apis*	*N. ceranae*	Neg
3	HIAs	*N. apis*	Neg	Neg
4	HIAs	Neg	Neg	Neg
5	HIAs	*N. ceranae*	Neg	Neg
6	LIAs	*N. apis*, *N. ceranae*	Neg	*N. apis*
7	LIAs	Neg	Neg	Neg
8	LIAs	Neg	Neg	Neg
9	LIAs	*N. apis*	Neg	Neg
10	LIAs	Neg	Neg	Neg

Although the difference of infection according to the origin of apiaries from HIAs and LIAs was not statistically significant (χ^2^ = 1.667, *p* = 0.200), a high risk of infection was observed for apiaries located in HIAs (*OR* = 6.00).

**Figure 2:**
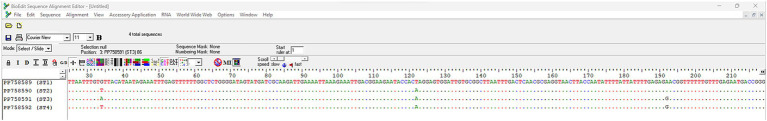
ClustalW multiple alignment of *Nosema ceranae* 16S rRNA sequence types showing single nucleotide polymorphisms in BioEdit software (version 7.7) (35).

All the 16S rRNA partial sequences of *N. apis* and *N. ceranae* herein found had 100% query coverage and 99.5–100% nucleotide identity with those available in the GenBank database. Whereas all sequences of *N. apis* were identical to each other, four different sequences types of *N. ceranae* (i.e., ST1 in pollen, ST2 in pollen, ST3 in bee, ST4 in pollen) were identified showing single nucleotide polymorphisms (SNPs) in the positions 34, 122, 192 (Figure 2). The computation of polymorphisms revealed high haplotype diversity (i.e., *Hd* = 1.000) and low nucleotide diversity (i.e., *Pi* = 0.00913) of *N. ceranae* 16S rRNA sequences from this study, with not statistically significant negative values of both Tajima’s D and Fu’s Fs tests (Table 3).

**Table 3:** Diversity and neutrality indices of *Nosema ceranae* 16S rRNA sequences (*n* = 4) from Southern Italy.

*S*	*Eta*	*k*	*Pi*	*Hn*	*Hd*	*πd*	Tajima’s D	*p*	Fu’s Fs	*p*
3	4	2.000	0.00913	4	1.000	0,03125	−0.78012	>0.1	−0,18,013	>0.1

*S*, Polymorphic (segregating) sites; *Eta*, Number of mutations; *k*, average pairwise nucleotide differences; *Pi*, nucleotide diversity; *Hn*, number of haplotypes; *Hd*, haplotype diversity; *πd*, variance of haplotype diversity; *p, p*-value.

## 4 Discussion

This study reports for the first time *N. apis* and *N. ceranae* infections in *A. mellifera* populations, pollen and honey, from apiaries of Southwestern Italy.

The high prevalence of *Nosema* spp. in the apiaries herein investigated (i.e., 60%) indicates a wide spread of infection in the study area, in agreement with the national average (30–69%) (36), and with the last data available from Central (63.2%) (46), Northern (42.9–54.5%) (47, 48) and Southeastern Italy (100%) (15). The finding of *N. apis* is of concern due to its high pathogenic potential (49, 50) and the fact that this species has been previously reported only in 2010 in Northern Italy (36, 51), and recently in the Southeast (15). This study also confirms the occurrence of *N. ceranae* in the south (15, 36), as already outlined in areas of Northern (47, 48) and Central Italy (46, 52).

As well as in bees, the finding of *N. apis* and *N. ceranae* in honey and pollen samples, respectively, is not surprising given that the small size of their spores (2–6 μm) (53, 54), allow them to be transferred as wind-dispersed bioaerosols from the air to different surfaces, including pollen and honey (14, 55). Another potential route of transmission may occur *via* bee feces contaminated with spores, which are usually left by bees near the apiary, carried by the wind to the flowers, and then collected again by bees during pollination (56). In addition, feeding bee colonies with infected honey and pollen may represent a further risk for the transmission of *Nosema* spp. infections (57, 58).

The signs of infection herein observed, such as high mortality and low honey production, are in accordance with the clinical picture of nosemosis (13, 15, 52, 59).

Although no statistically significant difference between apiaries in HIAs and LIAs is observed (*p* = 0.200), a higher risk of infection is emerged in HIAs than LIAs (*OR* = 6.00), suggesting that environmental aspects of HIAs (i.e., pollution, intense land use, low air quality, high bee density) (38) are implicated in the epidemiology of *Nosema* spp., as well as in the overall welfare of the bee industry. This hypothesis finds support in studies indicating that environmental stressors may increase the virulence of *N. ceranae* (60) and influence the bee microbiota, thus altering the immune system (61) and susceptibility to pathogens (62–65). Accordingly, further studies for assessing the correlation between apiary typology and susceptibility to *Nosema* spp. infections are required.

The presence of SNPs in all the 16S rRNA sequences of *N. ceranae* from pollen (i.e., ST1, ST2, ST4) and bees (i.e., ST3) of this study, could be due to a wide genetic variability of this pathogen, in accordance with previous studies showing the presence of several sequence types of this species (51, 66–70). The high haplotype diversity and low nucleotide diversity of *N. ceranae* sequences herein found are indicative for a rapid demographic expansion of this microsporidian. In accordance, although not statistically significant, the negative values of Tajima’s D and Fu’S Fs suggest an excess of rare polymorphic sites which are typical features of both recent population expansion and presence of rare haplotypes compared to what is expected under neutrality, pointing to past bottleneck and/or purifying selection events (43, 44).

Finally, the fact that nosemosis is no longer a notifiable disease (71) underlines the importance of epidemiological investigations as the only way to highlight the presence of *Nosema* spp. in apiaries (15). Accordingly, future large-scale surveys are needed to investigate the circulation of *Nosema* spp. in other areas of Italy and the potential implications to the honey bee industry. At the same time, epidemiological and pathogenetic insights are required on little-investigated trypanosomatids (e.g., *Lotmaria passim*) circulating in bee colonies (72) and honey with a high percentage (80%) of co-occurrence with *N. ceranae* microsporidia (55).

## Data Availability

The datasets presented in this study can be found in online repositories. The names of the repository/repositories and accession number(s) can be found at: https://www.ncbi.nlm.nih.gov/genbank/, PP758584; https://www.ncbi.nlm.nih.gov/genbank/, PP758589; https://www.ncbi.nlm.nih.gov/genbank/, PP758590; https://www.ncbi.nlm.nih.gov/genbank/, PP758591; https://www.ncbi.nlm.nih.gov/genbank/, PP758592.

## Footnotes

^1^ http://blast.ncbi.nlm.nih.gov/Blast.cgi
