# Supplementary material for: Bees on the run: Nosema spp. (Microsporidia) in Apis mellifera and related products, Italy
Source: Front Vet Sci. 2025 Jan 6;11:1530169. doi: 10.3389/fvets.2024.1530169 (PMC11743364; doi:10.3389/fvets.2024.1530169)

Evolutionary analysis by Maximum Likelihood method

The evolutionary history was inferred by using the Maximum Likelihood method and Hasegawa-Kishino-Yano model [1]. The tree with the highest log likelihood (-571.21) is shown. The percentage of trees in which the associated taxa clustered together is shown next to the branches. Initial tree(s) for the heuristic search were obtained automatically by applying the Maximum Parsimony method. A discrete Gamma distribution was used to model evolutionary rate differences among sites (5 categories (+*G*, parameter = 0.6210)). The tree is drawn to scale, with branch lengths measured in the number of substitutions per site. This analysis involved 19 nucleotide sequences. There were a total of 219 positions in the final dataset. Evolutionary analyses were conducted in MEGA X [2]. Sequences obtained in this study are reported in bold black.

1. Hasegawa M, Kishino H, Yano T. Dating the human-ape split by a molecular clock of mitochondrial DNA. J Mol Evol. (1985) 22:160-174. doi: 10.1007/BF02101694
2. Kumar S, Stecher G, Li M, Knyaz C, Tamura K. MEGA X: Molecular Evolutionary Genetics Analysis across computing platforms. Mol Biol Evol (2018) 35:1547-1549. doi: 10.1093/molbev/msy096

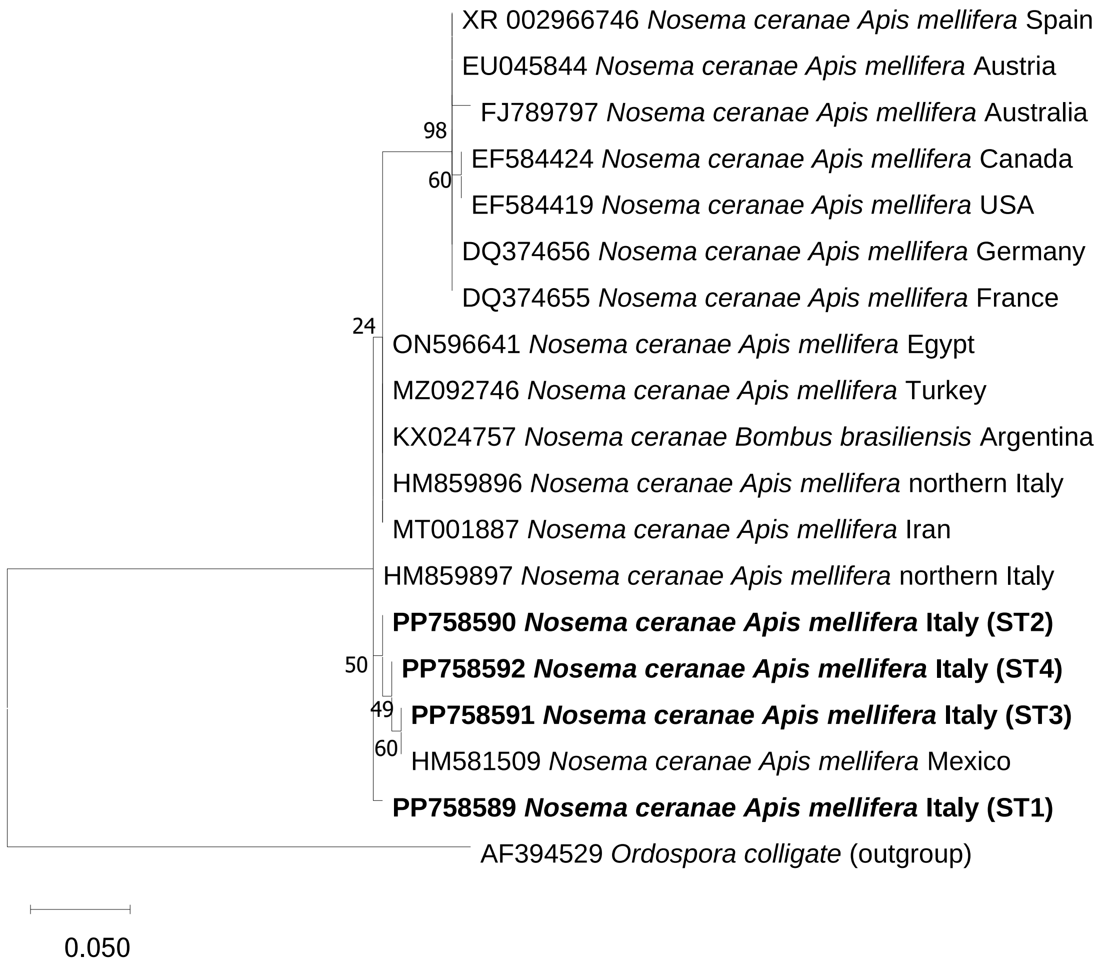

Supplement: Supplementary file 1 [file Data_Sheet_1.pdf]
